# Evolving concepts in extra-articular hip pathology

**DOI:** 10.1093/jhps/hnv018

**Published:** 2015-06-06

**Authors:** Hal D. Martin

**Affiliations:** Hip Preservation Center Baylor University Medical Center Dallas, USA E-mail: haldavidmartin@yahoo.com

The ISHA Annual Meeting in Rio de Janeiro 2014 provided fresh insight into extra-articular hip pathology and evolving treatments. The Instructional Course Lecture -2 was given the opportunity to present the lecture series in a published format in the *Journal for Hip Preservation Surgery*. Extra-articular procedures involve the osseous, capsulolabral, musculotendinous, neurovascular and kinematic chain requiring a comprehensive physical examination, diagnostic and treatment strategies. One component can affect each of the others with ascending or descending pathology. This complexity of pathology requires a systematic examination, radiographic interpretation and a thoughtful multistructured treatment program. The extra-articular pathology mentioned within this special series provides an endoscopic approach to more commonly perform open procedures. Endoscopic or arthroscopic diagnosis allows for improved visualized assessment of the complete anatomy along with the complexity of the multistructured presenting pathology. The following series addresses osseous endoscopic techniques including concepts of endoscopic-assisted Periacetabular Osteotomy, femoral derotational osteotomy, hamstring repair, deep gluteal space and intrapelvic sciatic entrapment. As the field of endoscopy and arthroscopy around the hip evolves, it is important to recognize the advancements and understanding of the anatomy, biomechanics, clinical examination and diagnostic strategies and treatment of each structure around the hip.

## CONFLICT OF INTEREST

None declared.

